# Significant increase in cultivation of *Gardnerella vaginalis*, *Alloscardovia omnicolens*, *Actinotignum schaalii*, and *Actinomyces* spp*.* in urine samples with total laboratory automation

**DOI:** 10.1007/s10096-018-3250-6

**Published:** 2018-04-13

**Authors:** Sabrina Klein, Dennis Nurjadi, Susanne Horner, Klaus Heeg, Stefan Zimmermann, Irene Burckhardt

**Affiliations:** Department of Infectious Diseases, Medical Microbiology, University Hospital Heidelberg, Heidelberg, Germany

**Keywords:** Laboratory automation, Urine, *Gardnerella vaginalis*, *Actinotignum schaalii*, *Alloscardovia omnicolens*, *Actinomyces* spp*.*

## Abstract

While total laboratory automation (TLA) is well established in laboratory medicine, only a few microbiological laboratories are using TLA systems. Especially in terms of speed and accuracy, working with TLA is expected to be superior to conventional microbiology. We compared in total 35,564 microbiological urine cultures with and without incubation and processing with BD Kiestra TLA for a 6-month period each retrospectively. Sixteen thousand three hundred thirty-eight urine samples were analyzed in the pre-TLA period and 19,226 with TLA. Sixty-two percent (*n* = 10,101/16338) of the cultures processed without TLA and 68% (*n* = 13,102/19226) of the cultures processed with TLA showed growth. There were significantly more samples with two or more species per sample and with low numbers of colony forming units (CFU) after incubation with TLA. Regarding the type of bacteria, there were comparable amounts of Enterobacteriaceae in the samples, slightly less non-fermenting Gram-negative bacteria, but significantly more Gram-positive cocci, and Gram-positive rods. Especially *Alloscardivia omnicolens*, *Gardnerella vaginalis*, *Actinomyces* spp*.*, and *Actinotignum schaalii* were significantly more abundant in the samples incubated and processed with TLA. The time to report was significantly lower in the TLA processed samples by 1.5 h. We provide the first report in Europe of a large number of urine samples processed with TLA. TLA showed enhanced growth of non-classical and rarely cultured bacteria from urine samples. Our findings suggest that previously underestimated bacteria may be relevant pathogens for urinary tract infections. Further studies are needed to confirm our findings.

**Electronic supplementary material:**

The online version of this article (10.1007/s10096-018-3250-6) contains supplementary material, which is available to authorized users.

## Introduction

Total laboratory automation (TLA) is well established in laboratory medicine and clinical chemistry. There have been numerous reports of improvements regarding quality of results, lower turnaround times, and higher efficacy. In diagnostic microbiology, automation has not been widely used. There are three platforms that offer a complete laboratory automation system: BD Kiestra, Copan Diagnostics WASPLab, and I2A Recitals [1, 2].

Lab automation platforms offer all steps of specimen processing, starting with inoculation of media, transport of media to incubators, standardized incubation times, digital photography of culture plates, and reading and processing of cultures for identification and antimicrobial susceptibility testing. Lower time to result (TTR) and a higher quality are expected, but still there are few studies published [3, 4].

Inoculation of urine samples with InoqulA (BD Kiestra) improved standardization compared to conventional microbiology [5] and produced more accurate results compared to WASP instrument [6]. As incubation time can be reduced, shorter TTR has been reported [3, 5, 7–9].

In the present study, we analyzed in total 35,564 urine samples in routine microbiological diagnostic pre- and with TLA. The main aim of this study is to investigate the performance of TLA in terms of culture quality and recovery of bacteria.

## Materials and methods

A total of 35,564 urine samples, 16,338 pre-TLA, and 19,226 samples with TLA, were analyzed in the study period. Replicate samples from the same patient were included in the study.

### Processing of urine samples pre-TLA

In the first study period, 10 μl urine samples were inoculated on Columbia agar with 5% sheep blood (BD Diagnostics, Heidelberg, Germany) and CPS (bioMérieux, Marcy l’ Etoile, France) plates with Previ Isola streaking machine (bioMérieux, Marcy l’ Etoile, France), transported manually and incubated overnight in 5% CO_2_ (Columbia agar) and in ambient air (CPS) at 37 °C. Plates were assessed for growth, species identification, and antibiotic susceptibility testing on the next working day. Species identification was performed by conventional microbiological methods (bench-top tests) and MALDI-TOF (matrix-assisted laser desorption-ionization time of flight) mass spectrometry (MS) (Bruker Diagnostics, Billerica, MA) or Vitek2 (bioMérieux, Marcy l’ Etoile, France).

### Processing of urine samples with BD Kiestra TLA system

Samples processed by the TLA were inoculated using the InoqulA module of the BD Kiestra TLA System. Briefly, 10 μl of the urine samples were inoculated and streaked using streaking pattern 4 on Columbia agar with 5% sheep blood (BD Diagnostics, Heidelberg, Germany) and CPS (bioMérieux, Marcy l’ Etoile, France). Immediately after inoculation, the plates were transferred automatically to the CO_2_ incubator for blood agar and ambient air incubator for CPS plates, where they were incubated for 24 h prior to imaging and reading. Quantification was performed by a standardized quantification scheme, which was validated with standardized inoculum. Species identification was determined using MALDI-TOF (Bruker Diagnostics, Billerica, MA) or Vitek2 (bioMérieux, Marcy l’ Etoile, France). Due to workflow issues, microbiological tests like catalase, coagulase and oxidase were not used when working with BD Kiestra TLA; more isolates were identified by MALDI-TOF MS and Vitek2. For details on number of performed IDs see supplemental material Figure 1.

### Interpretation criteria

The interpretation criteria did not change during the two time periods. For details, see supplemental material.

### Data analysis

Data were extracted from the laboratory information system (SWISSLAB, Roche Diagnostics, Mannheim, Germany). Data were analyzed using STATA 13 (StataCorp, USA) and Prism v5.0 (La Jolla, USA). Odds ratio (OR) and confidence interval (95% CI) were calculated using pre-TLA samples as reference. Culture-positive samples were compared to the number of samples analyzed, while for mono- and polymicrobial samples, all culture-positive samples were used as reference. For identified bacterial species, *E. coli* was used. Wilcoxon rank-sum test was used to analyze time-to-report (TTR). *P* values were calculated using the *χ*^2^ test and a *p* value ≤ 0.05 was considered statistically significant. Figures were generated with GraphPad Prism (La Jolla, USA).

## Results

Between July and December 2015, 16,338 and in the same time period in 2016, 19,226 urine samples were sent for microbiological analysis. Exact number and type of urine samples are provided as supplemental material 2.

Pre-TLA, 62% (10,101/16338) specimens were culture-positive. Among these, 4722 samples (47%) showed growth of one organism, 2743 (27%) with two, and 2636 (26%) with more than two bacterial species. With TLA, 13102 of 19,226 (68%) samples were positive. One species was grown in 5419 (41%), two in 2737 (20%), and more than two in 4946 (38%) samples. For statistical analyses, see Table 1.

**Table 1:** Culture-positive urine samples pre-TLA and with TLA by number of recovered bacterial species

	pre-TLA		TLA				
	*n*	%	*n*	%	OR	95% CI	*p*
Culture-positive samples	10,101	62	13,102	68	1.32	1.26–1.38	*p* < 0.001
1 species/sample	4722	47	5419	41	0.80	0.76–0.85	*p* < 0.001
2 species/sample	2743	27	2737	21	0.71	0.67–0.75	*p* < 0.001
> 2 species/sample	2636	26	4946	38	1.72	1.62–1.82	*p* < 0.001

OR with 95% CI and *p* value of specimen with one, two or more species per sample with TLA as compared to pre- TLA

*TLA*, total laboratory automation; *OR*, odds ratio; *95% CI*, 95% confidence interval

Patient characteristics regarding age, sex, and in- or outpatient status was similar between the two time periods. For details, see supplemental material Table 1.

Low numbers of CFU/ml (10^2^ and 10^3^ CFU/ml) were found more often in the urine samples processed with TLA. The number of specimen with 10^4^ and 10^5^ CFU/ml was comparable between the two time periods. With TLA, there were less samples with > 10^5^ CFU/ml. For details and statistics, see supplemental material, Table 2.

The cultural detection of Enterobacteriaceae and Gram-positive cocci was comparable between the two groups, while detection of non-fermentative Gram-negative bacteria was slightly lower with TLA. Meanwhile twice the amount of Gram-positive rods was detected with the TLA (254 vs 547, OR 2.0, 95% CI 1.72–2.36).

**Fig. 1:**
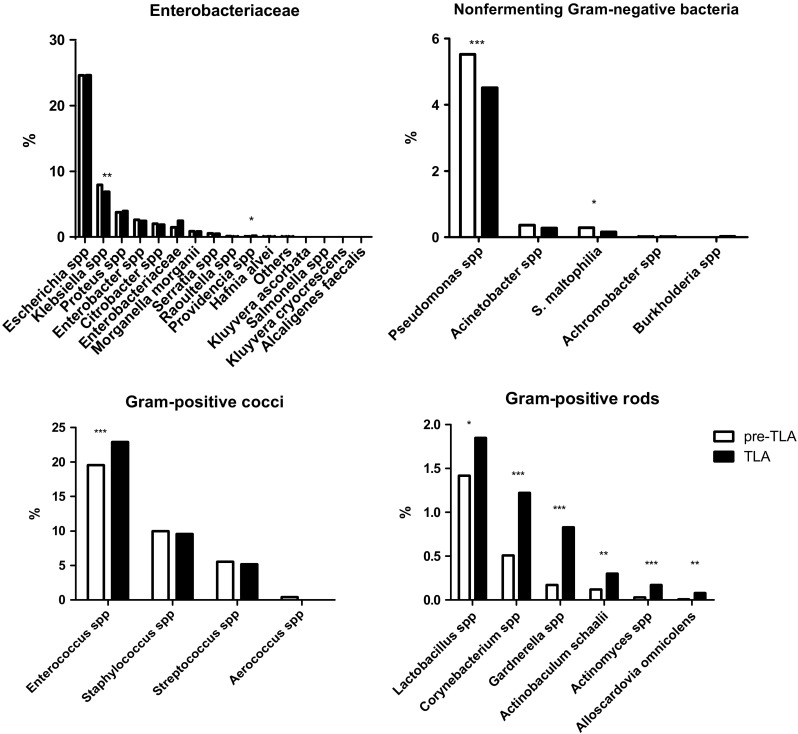
Percentage of all identified species during the study period. *p < 0.05, **p < 0.01, ***p < 0.001

Among the Enterobacteriaceae, *Klebsiella* spp*.* were isolated less and *Providencia* spp*.* more often with TLA. There were significantly less *Pseudomonas* spp*.* isolated as compared to the pre-TLA period. Of all Gram-positive cocci identified, *Enterococcus* spp*.* were significantly more frequent with TLA. Surprisingly, the cultivation of Gram-positive rods like *Lactobacillus* spp*.*, *Corynebacterium* spp*.*, *Gardnerella* spp*.*, *Actinotignum (Actinobaculum) schaalii*, *Actinomyces* spp*.*, and *Alloscardovia omnicolens* were significantly more frequent compared to the pre-TLA period (Fig. 1 and Table 2).

**Table 2:** Bacterial species cultured pre-TLA and with TLA

	pre-TLA	TLA	OR	95% CI	*p*
Enterobacteriaceae	4902	5187			
* Escherichia coli*	2727	2935	1.0	n/a	n/a
* Klebsiella* spp	880	821	0.87	0.78–0.97	*p* = 0.01
* Providencia* spp	10	23	2.14	0.96–5.04	*p* = 0.041
Non-fermenting Gram-negative bacteria	688	601			
* Pseudomonas* spp	612	538	0.82	0.72–0.93	*p* = 0.002
* S. maltophilia*	32	19	0.55	0.29–1.01	*p* = 0.038
Gram-positive cocci	3939	4657			
* Enterococcus* spp	2168	2730	1.17	1.08–1.26	*p* < 0.001
Gram-positive rods	254	547			
* Actinomyces* spp	3	20	6.19	1.83–32.56	*p* < 0.001
* Corynebacterium* spp	57	145	2.36	1.72–3.28	*p* < 0.001
* Gardnerella vaginalis*	19	99	4.88	2.93–8.39	*p* < 0.001
* Lactobacillus* spp	157	221	1.31	1.05–1.62	*p* = 0.013
* Alloscardovia omnicolens*	1	10	9.29	1.32–403.16	*p* = 0.01
* Actinobaculum schaalii*	13	36	2.57	1.33–5.29	*p* = 0.003
Others	4	16			

Number of identified bacterial species giving statistically significant results. OR with 95% CI and *p* value

*TLA*, total laboratory automation; *OR*, odds ratio; *95% CI*, 95% confidence interval; *n/a*, not applicable

Analyzing the urine samples with Gram-positive rods further, 25% (*n* = 25 of 99) of all specimens with growth of *Gardnerella vaginalis* came from kidney transplant recipients. Sixteen of the 36 (44%) samples with *Actinobaculum schaalii* were sent by the Department of Urology. Samples with *Alloscardovia omnicolens*, *Actinomyces* spp*.*, and *Corynebacterium* spp*.* were sent from all kind of patients throughout the hospital.

As we included all samples sent during the study period with repeated samples from patients, we wanted to verify that this is not a bias. In the pre-TLA period, *G. vaginalis* was isolated from 18 patients and with TLA from 84 patients. *A. omnicolens* was grown from samples of two patients pre-TLA and of nine patients with TLA. *A. schaalii* was isolated from 12 and 34 patients and *Actinomyces* spp*.* from 3 and 20 patients pre-TLA and with TLA, respectively.

**Fig. 2:**
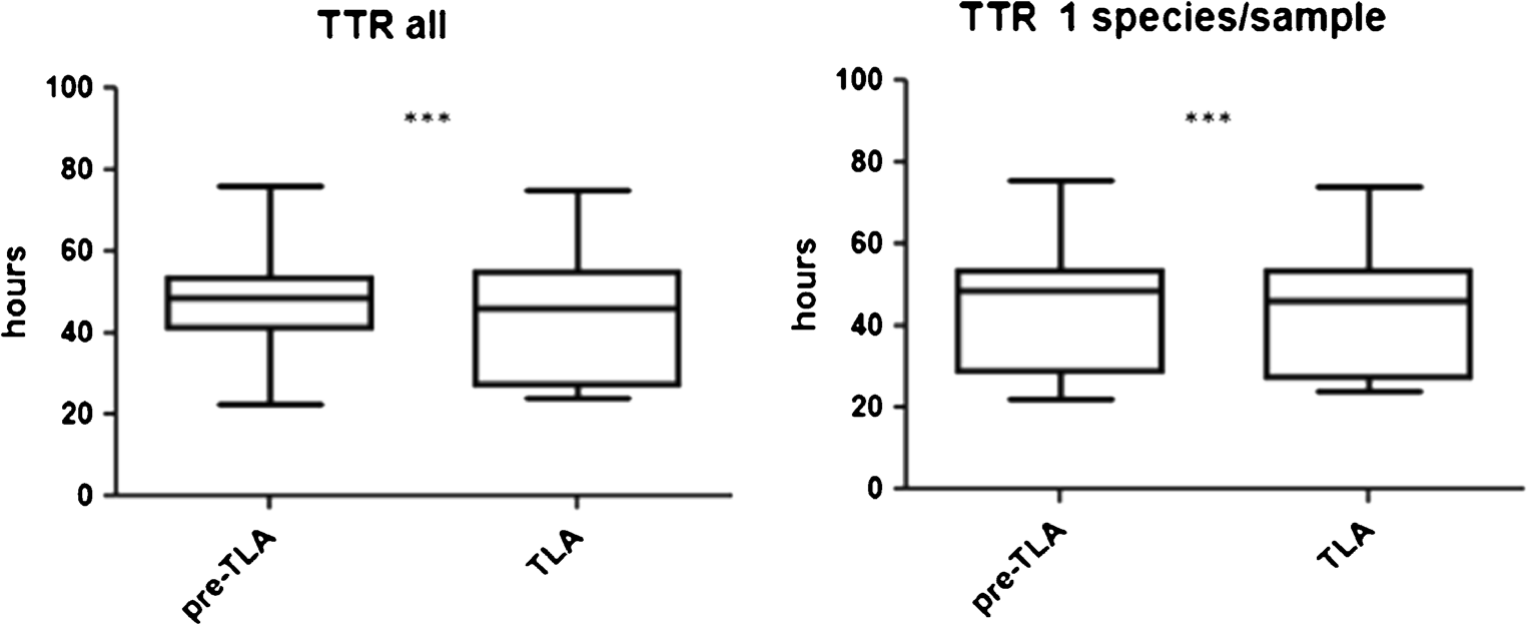
Time to result (TTR) of culture-positive samples. ***p < 0.001

The mean time to result of all culture-positive samples with TLA was 48.66 h compared to 49.98 h pre-TLA (*p* < 0.001). For samples with one species per sample, mean TTR with TLA was 1.5 h earlier than without TLA (47.94 and 49.45 h, respectively, *p* < 0.001) (Fig. 2). For samples with more than two species, TTR was 34.37 h with TLA and 35.14 h pre-TLA.

## Discussion

In the present study, a large number of urine samples have been analyzed in two comparable time periods. In the first time period from July to December 2015, 16,338 samples were analyzed of which 62% were culture-positive pre-TLA. During the second time period from July to December 2016, 19,226 samples were sent for analysis, and 68% of these showed growth of bacterial species. The time periods of July to December were chosen to avoid season-dependent changes. Due to the large number of urine specimen analyzed, it was not possible to compare each sample directly by parallel cultivation with and without TLA. Therefore, we cannot exclude a change in bacterial composition of urine samples in general, although this is not likely. In 2016, 48.8% of the samples were midstream urine while in 2015, 45.6%. Catheter urine was 34.3 and 37% of samples, respectively (see supplemental material). For both midstream and catheter urine samples, processing with TLA resulted in more culture-positive samples. Midstream and catheter urine taken together represent 83.1 and 82.7% of specimen sent for analyses. Thus, the higher recovery of bacterial species may not be attributes to a change in type of samples analyzed. Other studies could show high reproducibility and higher recovery of bacterial species by TLA compared to manual methods [5], which is in line with our findings.

### Streaking pattern may influence colony counting

For the pre-TLA processed urine samples, streaking was done by Previ Isola. The streaking pattern differs substantially from that used by TLA, as the Previ Isola uses a comb and streaks in a circle-like manner. With TLA, a meander-like pattern was used. For both type of systems, standardized inoculated samples were used to set up and validate a reading standard regarding the colony count before routine samples were analyzed. However, the differences in determining colony count may also be influenced by the different streaking patterns. Especially low colony counts of < 10^3^/ml were found more often with TLA. As low colony counts of 10^3^/ml and below are usually regarded as contamination with periurethral flora in midstream and catheter urine samples [10, 11], it is not a substantial issue.

### Differences in workflow

Due to workflow and logistic issues with TLA, less conventional tests like catalase, coagulase, or oxidase were used. Consequently, MALDI-TOF was used more often for species identification (see supplemental material, Figure 2). The identification with MALDI-TOF is more accurate than Vitek2, so there may be identification bias at the species level. The bacteria that are usually identified with catalase and coagulase are Staphylococci, and these were congruent with both identification methods. Moreover, the proportion of Staphylococci identified has not changed in both time periods. Oxidase is predominantly used to identify *Pseudomonas* spp*.* and other non-fermenting Gram-negative bacteria in urine samples. The proportion of non-fermenting Gram-negative bacteria and especially *Pseudomonas* spp*.* in the samples processed with TLA is significantly lower. The reason is not obvious, as other slow growing bacteria are identified more often, so the incubation time should be long enough. Part of the results may therefore be explained by the change in identification methods. Another point is the higher rate of polymicrobial samples. It is possible that *Pseudomonas* spp*.* are found more often in samples that are polymicrobial, but were not recognized as such in the pre-TLA period.

### Enhanced recovery of Gram-positive bacteria with TLA

Especially Enterococci and Gram-positive rods were isolated more frequently with TLA (Fig. 1 and Table 2). Enterococci are a frequent cause of urine tract infections. Their cultivation does not need special media or conditions, so they should be isolated in comparable frequency with both methods. Time to inoculation may play a role in these cases, as with TLA samples are processed faster and continuously throughout the working hours.

*Actinomyces* spp*.* are not described as uropathogenic so far. Due to the low numbers in the present study, we cannot conclude on clinical relevance of these bacteria in urine samples.

*Corynebacterium* spp*.* are known as commensal bacteria on skin and mucosal tissues. *C. urealyticum* has been described as an uropathogen [12], but only two of the isolated corynebacteria in our study were *C. urealyticum*. The frequency of *C. urealyticum* in general is low, but it may be up to 10% in kidney transplant recipients [13]. Although a large number of these patients is treated in our hospital, the amount of *C. urealyticum* infections seems to be lower than expected. From the literature, cultivation up to 48 h is recommended, which may explain the low number of *C. urealyticum* isolated here.

*Lactobacillus* spp*.* are part of the mucosal flora especially in women and is not regarded as a pathogenic bacterium. It may be found in urine samples that were contaminated with vaginal flora. On the other hand, there are few reports about the significance of *Lactobacillus* spp*.* as a cause of UTI [14, 15]. Evidence is still lacking, one reason may be the insufficient cultivation of urine samples and subsequent low recovery of this bacteria.

*Gardnerella vaginalis* has been reported to be underestimated as a cause of urinary tract infections [16–18]. In our study, the enhanced incubation and immediate inoculation of media may have supported growth of *G. vaginalis*. Twenty-five percent of samples with *G. vaginalis* were recovered from kidney transplant recipients. Thus, the role in immunocompromised patients needs to be investigated further.

*Alloscardovia omnicolens* has been described as a rare cause of UTI [19, 20]. *A. omnicolens* was isolated from ten samples, all sent from patients of the Department of Urology and Nephrology. The relevance remains unclear as analyses of more cases involving this pathogen are needed.

*Actinotignum (Actinobaculum) schaalii* has been reported as a cause of UTIs mostly in the elderly or in patients with underlying urologic conditions [21–24]. Consistent with this observation, 30% (16/36) of samples with *A. schaalii* were sent from the Department of Urology.

### Polymicrobial samples were more frequent with TLA

Significantly more samples showed growth of more than one bacterial species, which has been reported previously [7]. These specimen are classically regarded as contaminated by vaginal or periurethral microbiota [10]. With regard to sample types or patient characteristics, no relevant differences between the two time periods could be observed. One recent review posed the question whether these samples are rather a polymicrobial UTI than a contamination [12]. Especially with regard to the growing number of immunocompromised patients or patients with altered urinary tracts, the current diagnostic algorithms may not be adequate as they do not consider the possibility of polymicrobial UTIs in highly susceptible patients like solid organ/stem cell recipients or patients with special urologic conditions. Furthermore, the clinical relevance of Gram-positive rods isolated frequently with TLA in the present study may have been underappreciated due to insufficient cultivation conditions.

### Time to result (TTR)

Previous studies suggested earlier growth in microbiological samples when processed with TLA and therefore resulted in shorter TTR [3, 7, 8]. In the present study, the mean time to result for all culture-positive samples was 48.66 h with TLA and 49.98 h pre-TLA. If one species was grown, TTR was reduced by 1.5 h from 49.45 to 47.94 h (Fig. 2). The difference was even smaller for samples with more than two species (34.37 vs 35.14 h, respectively). The impact of reduction of TTR by 1.5 h seems too low to have a relevant and significant impact on patient care and management. Therefore, there is still potential to decrease the TTR further by longer reading times, earlier, and 24/7 processing of cultures. It has been reported than incubation time may be reduced to 14 h [7] and the time to report may then dramatically decrease. This may then improve the early optimal antibiotic treatment. On the other hand, the shorter incubation time could reduce the recovery of slow growing species like *Gardnerella vaginalis*, *Alloscardovia omnicolens, Actinotignum schaalii*, or *Corynebacterium* spp. Further reduction of incubation times should therefore be considered with caution. Another option would be the implementation of two reading time points, to early detect growth but also not miss slow growing bacterial species.

## Conclusion

Taken together, we provide the first analysis of a large number of urine specimen in Europe processed with TLA. Recovery of Enterobacteriaceae is comparable with both methods. TLA favors growth of low colony counts and polymicrobial samples as well as Enterococci and Gram-positive rods, especially *G. vaginalis*, *A. schaalii*, *A. omnicolens*, *Actinomyces* spp*.*, *Corynebacterium* spp*.*, and *Lactobacillus* spp. Clinical significance of these results, particularly in highly susceptible patients, needs to be analyzed further.

## Electronic supplementary material

ESM 1 (DOCX 77 kb)
