# Supplementary material for: Significant increase in cultivation of Gardnerella vaginalis, Alloscardovia omnicolens, Actinotignum schaalii, and Actinomyces spp. in urine samples with total laboratory automation
Source: Eur J Clin Microbiol Infect Dis. 2018 Apr 13;37(7):1305–11. doi: 10.1007/s10096-018-3250-6 (PMC6015101; doi:10.1007/s10096-018-3250-6)
Supplement: Supplementary file 1 — (DOCX 77 kb) [file 10096_2018_3250_MOESM1_ESM.docx]

**Supplemental material**

**Interpretation criteria for urine samples**

Columbia blood agar plates and CPS plates were inspected for bacterial growth after the indicated time point as described in Materials and Methods. If no growth was detected, the specimen was considered and reported as culture negative. In case of visible growth, quantification was performed using a quantification scheme that was validated with standardized inoculum. In case of more than two different colony morphologies present on the agar plate, the sample was considered and reported as polymicrobial without further identification of the bacterial species; regardless of the CFU/ml. In case of one or two bacterial species, isolates with 10^3^ or more CFU/ml were identified further to detect clinically relevant pathogens. In both periods, identification of *Escherichia coli* and *Enterococcus spp* was done by color and morphology on the CPS plate as described by the manufacturer. In the pre-TLA period, catalase, coagulase, oxidase, MALDI-TOF MS or Vitek2 was used to identify the isolated pathogens further. In the TLA period, MALDI-TOF MS or Vitek2 was applied (for numbers of tests used, see supplemental material, figure 2)

**Fig 1**

Figure 1 Number of test performed when processing urine samples pre- TLA or with TLA. Pre- TLA. 2908 catalase, 581 coagulase, 628 oxidase tests, 3535 MALDI-TOF and 683 Vitek IDs were performed. With TLA, 73 catalase, 21 coagulase, 10 oxidase tests, 3094 MALDI-TOF and 2829 Vitek IDs were performed.

**Fig 2**

Figure 2 **a** Percentage of types of samples analyzed during the two study periods. 7455 midstream, 6054 catheter, 2477 urine without further specification, 44 puncture, 163 Uricult® dip slides and 145 bag urine samples were analyzed in the pre-TLA period. With TLA, 9386 were midstream, 6599 catheter, 3009 urine without further specification, 40 puncture urine samples, 78 Uricult® dip slides and 114 bag urine specimen were analyzed. **b** Percentage and type of culture positive of samples.

Table 1 Patient characteristics of culture positive samples.

|  | pre- TLA | TLA |
| --- | --- | --- |
| mean age (years±SD) | 56.7±22.2 | 58.0±21.9 |
| female (%) | 51.52 | 49.24 |
| outpatient (%) | 43.6 | 43.75 |

Table 1 patient characteristics of all culture positive samples.

Abbr.:TLA= total laboratory automation; SD= standard deviation.

Table 2 Amount of samples with 10^2^ - >10^5^ CFU/ml.

|  | pre-TLA | TLA | OR | 95% CI | p |
| --- | --- | --- | --- | --- | --- |
| 10^2^ CFU/ml | 1552 | 2591 | 1.42 | 1.33-1.52 | p<0.001 |
| 10^3^ CFU/ml | 1699 | 2783 | 1.4 | 1.31-1.49 | p<0.001 |
| 10^4^ CFU/ml | 2632 | 3401 | 1.06 | 1.0-1.13 | p=0.033 |
| 10^5^ CFU/ml | 2335 | 2810 | 0.97 | 0.92-1.04 | p=0.55 |
| >10^5^ CFU/ml | 5538 | 5328 | 0.68 | 0.65-0.72 | p<0.001 |

Table 2 OR with 95% CI and p value of urine samples 10^2^->10^5^ CFU/ml with TLA as compared to the pre-TLA period

Abbr: TLA=total laboratory automation; OR = odds ratio; 95% CI = 95% confidence interval; n/a= not applicable.
